# Co-Developing a Culturally Responsive, Theory-Informed Dyadic Mind–Body Intervention to Improve Sleep and Wellbeing in People with Dementia and Their Caregivers in the UK

**DOI:** 10.3390/healthcare14030383

**Published:** 2026-02-03

**Authors:** Sunny H. W. Chan, Rosa Hui, Zehra Haq, Richard Cheston

**Affiliations:** 1 Centre for Health and Clinical Research, University of the West of England, Bristol BS16 1QY, UK; 2 Chinese Community Wellbeing Society, Bristol BS16 2QQ, UK; rosa@chinesecws.org.uk; 3 Dhek Bhal, Bristol BS5 0AX, UK; zhaq55@yahoo.co.uk; 4 School of Social Sciences, University of the West of England, Bristol BS16 1QY, UK; richard.cheston@uwe.ac.uk

**Keywords:** dementia, sleep disturbance, mind-body intervention, co-design, Intervention Mapping, Behaviour Change Wheel, ethnic minority

## Abstract

**Highlights:**

**What are the main findings?**

- A culturally grounded, theory-informed dyadic mind–body intervention was successfully co-developed with people with dementia, caregivers, and diverse community stakeholders, demonstrating high cultural resonance, acceptability, and feasibility.
- Behavioural analysis identified key determinants of engagement—such as cognitive load, cultural meaning, dyadic support, and practical barriers—which guided the selection of effective behaviour change techniques integrated into the final 8-week programme.

**What are the implications of the main findings?**

- The development work provides a strong foundation for conducting a feasibility trial to evaluate implementation, acceptability, and preliminary effects on sleep, caregiver wellbeing, and dyadic coping across diverse communities.
- The structured, culturally responsive co-design process offers a replicable model for developing future non-pharmacological interventions in dementia care, particularly where cultural adaptation and dyadic approaches are essential.

**Abstract:**

**Background:** Sleep disturbances are common in dementia and adversely affect both the person with dementia and their caregiver. Non-pharmacological options exist but are seldom dyadic or culturally tailored, limiting their reach and relevance across diverse communities. **Objective:** We aimed to co-develop DREAM (Dyadic Resilience, Engagement, Awareness & Mind–body intervention)—an 8-week dyadic mind–body programme (mindfulness + gentle Tai Chi) for improving sleep and wellbeing in people with dementia and their caregivers. **Methods:** The process was informed by Intervention Mapping (Stages 1–4) and underpinned by established behaviour change frameworks, including the Behaviour Change Wheel (BCW), the COM-B model (Capability, Opportunity, Motivation → Behaviour), and the Theoretical Domains Framework (TDF), to systematically identify determinants of engagement. Co-design involved dementia–caregiver dyads, Patient and Public Involvement (PPI) contributors, clinicians, mind–body practitioners, and community stakeholders. **Results:** The intervention was co-developed and culturally grounded through engagement with White British, Caribbean, Chinese, and South Asian communities. Participants reported high cultural resonance, endorsing DREAM’s concise practices, caregiver-supported home routines, and delivery in trusted community venues. Behavioural insights highlighted the importance of motivational framing (perceived dyadic benefits, cultural meaning), practical enablement (simplified guidance, prompts/cues, environmental restructuring), and caregiver facilitation to support adherence. **Conclusions:** DREAM demonstrates the practicability of using Intervention Mapping to co-develop a culturally responsive, theory-informed dyadic mind–body intervention for people with dementia and their caregivers. This groundwork supports progression to a feasibility trial focused on implementation processes and preliminary sleep and wellbeing outcomes.

## 1. Introduction

Sleep disturbances are among the most common and distressing symptoms of dementia, and have substantial implications for both individuals living with the condition and their caregivers [1,2]. In Alzheimer’s disease, poor sleep is associated with impaired clearance of amyloid-β, a neuropathological hallmark that accelerates disease progression [3,4]. Beyond biological mechanisms, sleep disruption contributes to fatigue, mood disturbance, and behavioural symptoms such as agitation and aggression [5,6,7], all of which heighten caregiver burden [8,9]. Caregivers themselves frequently experience poor sleep due to both the nocturnal disruptions of the person with dementia and the psychological and physical strain of caregiving [10,11,12,13]. Consequently, sleep problems in dementia are best conceptualised as a dyadic phenomenon—where a partner or co-residing caregiver is present—because disturbances impact both the individual with dementia and the wellbeing of their caregiver.

Current responses to sleep disturbance in dementia remain largely medical and reactive, despite Cochrane reviews highlighting the weak evidence for medication and the need for safe, sustainable, non-drug alternatives [14,15]. Mind–body approaches such as mindfulness, breathing exercises, and Tai Chi show promising benefits for sleep, stress, and overall wellbeing in older adults with dementia [16,17,18]. However, existing interventions rarely engage both the person with dementia and their caregiver concurrently, nor do they typically integrate multiple forms of support within a single programme.

Emerging evidence suggests that sleep disturbances and their consequences may be more pronounced among certain ethnic groups, particularly people from Black and South Asian communities. For example, studies show that non-Hispanic Black adults experience greater sleep discontinuity and poorer sleep quality than White adults, with associated impacts on memory performance [19] and increased vulnerability during stressful periods such as COVID-19 [20]. Research involving participants from South Asian and Black populations also reports shorter sleep duration and higher burdens of chronic conditions linked to poorer sleep [21]. Additionally, ethnic differences in modifiable health risk factors may further contribute to disparities relevant to dementia risk [22]. Yet little is known about how ethnically diverse communities perceive and manage sleep disturbance in dementia, and culturally tailored interventions remain scarce. In the UK, the number of individuals with dementia from minority ethnic backgrounds is predicted to increase substantially in the coming decades [23]. However, these groups are less likely to access dementia services, often doing so only in later stages or during crises [24]. Health inequalities—including stigma, structural inequities, and a lack of culturally appropriate care—further restrict access to timely and effective support [25].

Addressing these challenges needs a clear, structured, and theory-based approach. Sleep interventions for people with dementia are naturally complicated. They often include several components—such as environmental modifications, daily activity scheduling, relaxation, and caregiver support strategies—that need to working together to be effective, may need to support both the person with dementia and their caregiver, and must still work for individuals who do not have a care partner. In addition, interventions must be sensitive and responsive to diverse cultural and community contexts. The updated Medical Research Council (MRC) Framework for complex interventions emphasises systematic, theory-informed development that considers mechanisms of change, implementation processes, and equity from the outset [26]. However, existing caregiver sleep interventions remain small-scale, heterogeneous, and weakly theorised, highlighting the need for more structured designs [27]. Similarly, reviews of dyadic interventions in dementia underscore the importance of articulating behaviour-change mechanisms and accounting for contextual variability [28]. Behavioural science has begun to inform dementia sleep interventions, as exemplified by the DREAMS-START programme [29], which combined psychoeducation, routine optimisation, light exposure, physical activity, and structured caregiver support while examining mechanisms of change. However, these approaches have not yet been culturally adapted to meet the needs of communities at higher risk, nor designed to benefit both people living with dementia and their caregivers at the same time.

Building on this progress, the present study integrates behaviour-change principles with culturally resonant, embodied practices—including mindfulness, breathwork, and gentle Tai Chi–based movement. Accordingly, the DREAM (Dyadic Resilience, Engagement, Awareness & Mind–body intervention) programme was developed using Intervention Mapping (IM; Stages 1–4) [30,31] and the Behaviour Change Wheel (BCW) and COM-B (Capability, Opportunity, Motivation → Behaviour) model [32,33] to ensure theoretical rigour, systematic development, and stakeholder relevance. This study aimed to co-develop and culturally ground the DREAM programme through collaboration with people living with mild dementia and their caregivers from White British, Caribbean, Chinese, and South Asian communities, alongside input from healthcare professionals. To our knowledge, this is the first dyadic, culturally adapted mind–body sleep intervention for people with dementia in the UK, addressing both clinical and cultural dimensions of sleep and wellbeing in dementia care.

## 2. Materials and Methods

### 2.1. Setting and Participants

The study took place in a city in Southwest England and involved 12 dementia–caregiver dyads, drawn equally from four ethnic communities: White British (n = 3), Caribbean (n = 3), Chinese (n = 3), and South Asian (n = 3). The inclusion of 12 dementia–caregiver dyads was consistent with guidance for early-phase complex intervention co-development, which prioritises depth, theory refinement, and feasibility over statistical representativeness [26]. Qualitative saturation literature suggests that core experiential themes are typically identified within 6–12 cases, particularly in focused, theory-informed studies [34]. Equal representation of four ethnic communities (three dyads per group) followed principles of maximum variation sampling, ensuring balanced cultural input into intervention design rather than comparative inference [35]. This approach aligns with prior mind–body and integrative medicine intervention development studies and supports culturally responsive, dyadic intervention refinement.

The co-development process also included six Patient and Public Involvement (PPI) representatives and two Tai Chi and mindfulness practitioners with experience in culturally tailored practice. Within each community, we held two co-development consultation sessions followed by a pilot run of eight sessions of the co-designed DREAM programme, with both activities contributing directly to Intervention Mapping (IM) Stages 2–4. Through these sessions, contributors helped refine the programme’s content, delivery style, and cultural adaptations. Their involvement was essential to ensuring that the final intervention was both culturally relevant and practically feasible. Participants were identified and recruited through trusted local networks—including Voluntary, Community and Social Enterprise (VCSE) Sectors—to ensure meaningful representation across ethnic, linguistic, and socio-cultural backgrounds. This approach allowed a broad range of perspectives to inform the programme’s content, delivery, and acceptability, strengthening the credibility of the co-design process.

### 2.2. DREAM Development and Procedures (Structured by IM Stages)

#### 2.2.1. Design Framework

IM provided a clear, structured framework for developing DREAM in a systematic and co-produced way. Using the Behaviour Change Wheel (BCW) and COM-B (Capability, Opportunity, Motivation → Behaviour) frameworks helped identify key barriers and facilitators and guided the selection of practical behaviour-change strategies. This process ensured that DREAM was theory-driven, culturally grounded, and shaped directly by stakeholder experience, in line with best practice for developing complex health interventions [35,36]. Table 1 summarises the key development tasks.

#### 2.2.2. Stage 1: Needs Assessment

Guided by IM, Stage 1 was previously undertaken in an earlier phase of this research programme. The multi-source needs assessment identified priorities, contextual factors, and behavioural determinants for a dyadic mind–body intervention targeting sleep disturbances in dementia–caregiver dyads. A diverse working group—comprising dementia–caregiver dyads from White British, Caribbean, Chinese, and South Asian communities, alongside PPI contributors, mind–body practitioners, community leaders, and clinicians—collaboratively guided the process. Data from focus groups, stakeholder consultations, and a targeted literature review were integrated to refine intervention outcomes.

In that earlier phase, workshops and prioritisation exercises were conducted to identify and rank key outcome domains for the intervention. Participants independently rated the importance of potential targets, and group discussions were used to refine priorities for subsequent programme development. The BCW and COM-B framework were then applied to systematically identify behavioural determinants relevant to engagement with mind–body practices. Using structured prompts aligned with Capability, Opportunity, and Motivation domains, participants reported perceived barriers and facilitators to learning and sustaining sleep-related practices. These data were integrated with insights from focus groups and the targeted literature review to generate an initial problem analysis and needs statement. This process informed specification of the core outcomes and behavioural targets to be addressed in Stage 2 of programme development.

#### 2.2.3. Stage 2: Programme Outcomes and Change Objectives

Stage 2 involved defining programme outcomes, specifying performance objectives, and developing matrices of change objectives to guide intervention design. Within each community, a new group of dementia–caregiver dyads, PPI representatives, mind–body practitioners, and community stakeholders took part in a structured workshop to review and expand upon the findings from Stage 1. Participants engaged in facilitated discussions to identify priority outcomes for improving sleep and wellbeing, after which the research team translated these priorities into performance objectives describing the behaviours required of people with dementia, caregivers, and facilitators. These draft objectives were iteratively refined to ensure cultural relevance and feasibility.

To identify determinants influencing these behaviours, data from Stage 1 and workshop activities were analysed using the COM-B model, with further categorisation informed by the Theoretical Domains Framework (TDF v2) [37]. This process enabled systematic identification of Capability-, Opportunity- and Motivation-related barriers and facilitators. Performance objectives and corresponding determinants were then mapped onto intervention functions from the BCW and linked to candidate behaviour change techniques (BCTs). The resulting matrix of change objectives specified what needed to change, for whom, and through which mechanisms, forming the behavioural pathway guiding Stage 3 (see Table 2).

#### 2.2.4. Stage 3: Theory-Based Methods and Strategies

Stage 3 involved selecting BCTs and operationalising them into delivery strategies for the DREAM intervention (Table 2). Candidate BCTs were identified through the BCW, BCT Taxonomy [33,38], the Theory and Techniques Tool [39], and relevant dementia and mind–body literature. These were reviewed in another iterative workshop in each community with PPI contributors, caregivers, practitioners, and community stakeholders to determine theoretical relevance, cultural suitability, and practical feasibility, including suitability for individuals with differing levels of cognitive impairment.

**Figure 1:**
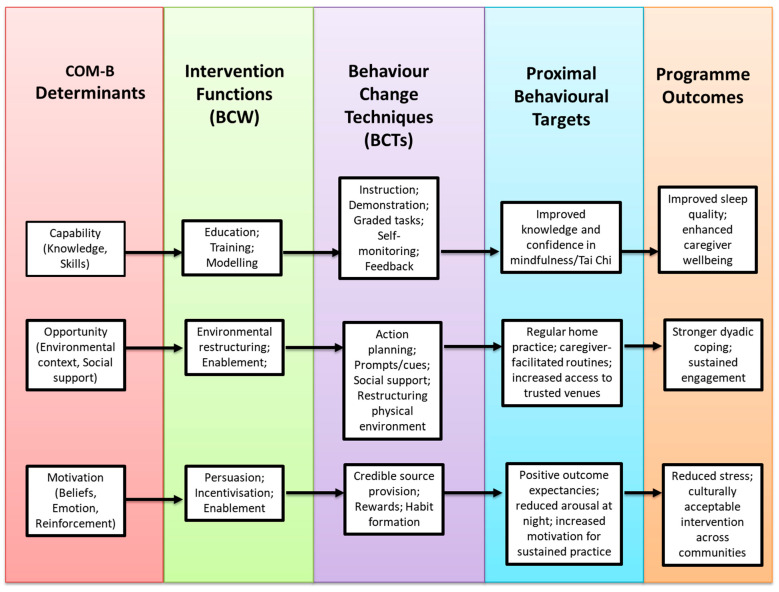
Logic model of change for the DREAM programme.

Selected BCTs were then translated into concrete delivery strategies through structured design activities. Particular attention was given to cognitive accessibility and intervention fidelity, resulting in delivery formats that incorporated simplified and repetitive instruction, a consistent session structure, and the use of multimodal cues (verbal guidance, live demonstration, and visual prompts). Practices were designed to be progressively scaffolded across sessions, allowing participants to engage at varying levels while maintaining the core intervention components. The dyadic format enabled caregivers to provide in-the-moment cueing, reassurance, and support, helping participants with greater cognitive impairment to remain engaged without disrupting group flow. A facilitator manual was developed to support fidelity and consistency. Finally, all techniques were mapped to COM-B determinants and proximal behavioural targets and organised within a logic model of change. This mapping clarified the intended pathways from intervention functions to behavioural change and the overarching outcomes of improved sleep, caregiver wellbeing, and dyadic coping (see Figure 1).

#### 2.2.5. Stage 4: Programme Design and Materials

Stage 4 involved translating the selected theory-based methods and strategies into a fully specified, culturally adapted intervention. Through an iterative co-design process with PPI contributors, caregivers, practitioners, and community stakeholders, the research team developed an eight-week dyadic programme integrating brief mindfulness practices and gentle Tai Chi-based movements, supported by caregiver-facilitated home practice. Session content, language, visuals, and examples were adapted to ensure relevance and accessibility across White British, Caribbean, Chinese, and South Asian communities.

Detailed delivery procedures were established to guide weekly session structure, progression of skills, and home-practice planning. A comprehensive resource package—including a Facilitator Manual, session outlines, simplified practice guides, culturally adapted handouts, visual aids, video links, and home-practice logs—was created to support consistent delivery. All materials were iteratively reviewed by PPI contributors and community stakeholders for clarity, cultural appropriateness, and usability. Programme components were systematically checked against the logic model of change to ensure alignment with identified determinants, behaviour change techniques and intended outcomes. This process produced a theory-driven, culturally responsive intervention ready for pilot evaluation.

### 2.3. Ethical Considerations

Ethical approval for the study was granted by the Faculty Research Ethics Committee at the University of the West of England on 11th February 2025 (UWE REC Ref No: 13515168), and all procedures adhered to the ethical principles outlined in the Declaration of Helsinki. Written informed consent was obtained from both individuals living with mild dementia and their caregivers prior to participation. All individuals with dementia were assessed as having the capacity to provide informed consent at the time of enrolment, in accordance with ethical guidance. Participants were given clear explanations of the study’s aims and procedures, along with opportunities to ask questions and withdraw at any time without consequence.

All data, including audio recordings and transcripts, were anonymised and securely stored on password-protected university servers accessible only to authorised members of the research team. Identifiable information was kept separate from research data, and pseudonyms have been used in all reports, publications, and dissemination materials to protect participant confidentiality. Data management adhered to institutional policies and research governance standards, with all audio recordings and transcripts retained for five years before secure destruction in line with data protection regulations. These procedures ensured that the study maintained full compliance with ethical principles for research involving vulnerable populations, promoting trust, respect, and participant safety across diverse cultural communities.

## 3. Results

### 3.1. Stage 1: Needs Assessment

The focus groups, stakeholder consultations [40] and targeted literature review [18,41]—reported previously—brought together dementia–caregiver dyads from White British, Caribbean, Chinese, and South Asian communities, alongside practitioners, clinicians, and community representatives. Across all groups, sleep disruption was consistently described as a strain on physical and emotional wellbeing, with clear implications for relationship dynamics and caregiving burden. Participants broadly viewed mind–body practices as acceptable and potentially beneficial but stressed the importance of approaches that were simple to follow, culturally relevant, and supportive of both members of the dyad, including through guided home practice.

The prioritisation and COM-B exercises further revealed shared concerns across communities, while also bringing important cultural nuances to light (see Table 3). Although all groups endorsed sleep and dyadic engagement as central targets, communities varied in how strongly they emphasised cultural fit, familiarity with mind–body approaches, and the practical constraints that might shape engagement. For example, cultural acceptability was particularly prominent among Chinese and South Asian participants, reflected also in their reports of lower familiarity with mind–body practices and greater language-related challenges. Caribbean and White British groups highlighted fewer cultural or linguistic barriers but raised issues related to transport, motivation, and competing responsibilities. Motivation-related facilitators, such as the cultural meaning of practices and perceived dyadic benefit, were widely endorsed, yet the extent of scepticism or stigma differed notably between groups. Taken together, these findings illustrate both common behavioural determinants and specific cultural considerations that informed the development of programme goals and pathways for subsequent IM stages.

### 3.2. Stage 2: Programme Outcomes and Change Objectives

A further 12 dyads from the four ethnic communities participated in IM Stages 2–4, together with PPI representatives, mind–body practitioners, and community stakeholders. Together, these groups took part in a structured prioritisation process using facilitated discussions, ranking exercises, and iterative feedback rounds. Through this consensus-building process, stakeholders reviewed the needs assessment findings and collectively identified the three outcomes that the programme should focus on. The final outcomes—(i) improved sleep quality, (ii) enhanced caregiver wellbeing, and (iii) strengthened dyadic coping and engagement—were therefore co-agreed. Agreement was reached when at least 80% of participants across all groups ranked an outcome as essential or high priority. These outcomes were theorised by the research team and the PPI contributors to yield short-term benefits (e.g., reduced stress, increased participation) and longer-term benefits (e.g., improved quality of life and reduced caregiver burden).

Four performance objectives were identified through a structured co-production process. These objectives were developed during facilitated workshops in which participants reviewed the prioritised outcomes and then worked together—using brainstorming, discussion, and ranking exercises—to specify what behaviours would be required for the DREAM programme to achieve its aims. The four agreed performance objectives were: (1) establishing consistent dyadic bedtime routines; (2) integrating mindfulness or Tai Chi into daily life; (3) reducing night-time stress; and (4) maintaining caregiver-supported home practice. Workshops highlighted the need for simplified movements, stepwise guidance, and accessible materials. To understand what might help or hinder these behaviours, we explored determinants—factors that influence whether a behaviour can occur. These determinants were generated through participant discussions and then coded using the COM-B model and mapped to the TDF for behavioural precision: cognitive and physical limitations and limited familiarity (Capability); trusted venues, flexible scheduling, culturally relevant materials, and caregiver support (Opportunity); and perceived dyadic benefit, sleep-related goals, and cultural resonance (Motivation). Despite overall enthusiasm, stigma and scepticism emerged as remaining barriers—particularly among some participants from Chinese and South Asian communities who expressed uncertainty about discussing sleep problems openly due to cultural norms around privacy, reluctance to burden others, and discomfort talking about personal or family difficulties. These concerns influenced motivation, highlighting the need for sensitive communication and normalisation within programme delivery. Together, these defined the behavioural pathway guiding Stage 3.

### 3.3. Stage 3: Theory-Based Methods and Strategies

Stage 3 refined a structured suite of theory-driven BCTs guided by COM-B and IM. Core BCTs included action planning, habit formation, self-monitoring, prompts and cues, demonstration and modelling, graded practice, social support, credible source provision, and environmental restructuring, with cultural adaptations such as using metaphors and imagery relevant to Chinese and South Asian traditions. BCTs were operationalised through practical strategies: structured home-practice schedules, practice logs, guided group demonstrations, visual prompts, culturally resonant materials, and dyadic support activities. Environmental restructuring was achieved through delivery in trusted community venues. These settings were important not only for their accessibility but also because participants associated them with cultural safety and familiar, trusted people. This increased comfort and willingness to engage, highlighting the value of locating interventions within community-based, culturally relevant spaces for effective service delivery.

Each BCT was mapped to Capability, Opportunity, and Motivation determinants and aligned with proximal behavioural targets (e.g., routine building, arousal reduction, dyadic support) and the overarching outcomes of improved sleep, caregiver wellbeing, and dyadic coping. The resulting guiding principles emphasised dyadic involvement, clear outcome setting, motivation-building, structured action planning, self-monitoring, regular review, problem-solving, and progressive independence. This process clarified how the intervention was expected to work and why specific components were necessary. Importantly, these mapped components were then brought together into a logic model of change linking determinants, intervention functions, BCTs, and outcomes, as developed jointly by the research team and stakeholders (see Figure 1).

### 3.4. Stage 4: Programme Design and Materials

Stage 4 resulted in the final specification of the DREAM intervention and the development of a comprehensive package of supporting resources. Building on the theoretical, behavioural, and cultural foundations established in earlier stages, this phase translated the identified methods and strategies into a fully developed, ready-to-deliver programme that was both theory-driven and community-informed, and explicitly designed to accommodate heterogeneity in cognitive ability among participants.

The DREAM programme was structured as an eight-week dyadic course integrating brief mindfulness practices, and gentle Tai Chi–based movement, reinforced by caregiver-facilitated home practice. Weekly sessions were delivered in trusted community venues and tailored with culturally relevant language, imagery, and examples to ensure accessibility and resonance across White British, Caribbean, Chinese, and South Asian communities. Each session followed a consistent and predictable format—combining check-in, guided practice, reflective discussion, and planning for home practice—to reduce cognitive load and support familiarity over time.

Content was introduced gradually, with mindfulness and Tai Chi elements simplified and repeated across weeks to support learning and retention. Participants were encouraged to engage flexibly (e.g., observing, mirroring, or partially participating) according to their cognitive capacity on a given day, while caregivers provided supportive cueing and reassurance as needed. Delivery of the intervention during this stage was undertaken by the first author, who has over 20 years’ experience in delivering mindfulness meditation and Tai Chi, ensuring sensitive pacing, adaptation, and fidelity to the intervention’s core components. This structured progression enabled participants to develop skills gradually, with each week reinforcing previous learning while introducing new practices to deepen self-awareness, balance, and relational attunement (see Table 4 for the detailed weekly programme outline).

A comprehensive package of resources was produced to support the intervention. These included:

- Facilitator Manual
- Simplified stepwise mindfulness and Tai Chi guides
- Picture guides, illustrated handouts and video links
- Home practice logs
- Feedback and outcome monitoring tools

These materials were made available to participants in both printed and digital formats, depending on individual preference and accessibility needs. To ensure inclusivity, all core resources—including handouts, instructions, and practice logs—were provided in English, Punjabi, Urdu, and Chinese, with video demonstrations recorded in multiple languages where possible.

All materials underwent multiple rounds of iterative review by PPI contributors and community stakeholders from White British, Caribbean, Chinese, and South Asian communities. Their feedback directly shaped the clarity, cultural appropriateness, and accessibility of each resource, for example:

- Language and Tone: PPI reviewers identified terms that felt too clinical or unfamiliar; these were replaced with simpler, everyday language. Instructions were rewritten to be shorter, more direct, and more supportive in tone.
- Cultural Framing: Contributors recommended using metaphors and imagery meaningful within their communities—for instance, linking slow breathing to “settling the heart” in Chinese contexts or emphasising “mutual care” and family interdependence in South Asian groups.
- Movement Adaptations: Tai Chi movements were simplified further after reviewers noted physical limitations among elders, and culturally respectful seating arrangements were suggested for group sessions.
- Translation Quality: Bilingual PPI contributors improved the accuracy and cultural nuance of corresponding translations, ensuring that mindfulness terms were familiar and appropriate for older adults.
- Visual Materials: Chinese and South Asian groups suggested adding more diverse illustrations showing people who “look like us,” which was implemented across handouts and slides.

Programme refinement ensured all elements aligned with the logic model and directly addressed behavioural, social, and motivational determinants identified earlier—such as reducing cognitive load, building confidence with unfamiliar practices, and ensuring delivery settings felt culturally safe and trusted. The resulting DREAM intervention is theory-driven, culturally responsive, and ready for pilot evaluation and scaling.

## 4. Discussion

The DREAM programme was developed using a rigorous, theory-informed, and community co-design approach based on the IM framework. This systematic process integrated empirical evidence, behavioural theory, and extensive public engagement, resulting in an intervention that is evidence-informed, culturally responsive, and feasible for implementation within community settings. The co-development process engaged people living with dementia, their caregivers, mind–body practitioners, clinicians, and community representatives from four ethnic groups, ensuring that DREAM reflects the lived realities, preferences, and barriers of its intended users.

### 4.1. Relational Dimensions of Sleep in Dementia Care

The theoretical grounding of DREAM aligns with research highlighting the multifaceted and relational nature of sleep disturbance in dementia. Sleep disruption affects physical health, emotional regulation, cognition, and relationships, with recent studies showing dyadic consequences for wellbeing, emotional attunement, and relationship quality [42]. Qualitative work similarly demonstrates how night-time disturbances contribute to cumulative strain within caregiving partnerships [43]. Responding to this evidence, DREAM adopts a dyadic, non-pharmacological framework targeting sleep quality, caregiver wellbeing, and dyadic coping. The programme’s cultural responsiveness further broadens its biopsychosocial scope, recognising the relational and contextual dimensions of care.

During the co-design process, stakeholders emphasised that mutual support, attunement, and shared meaning are central to effective dyadic interventions—findings that align with evidence showing that dyadic engagement enhances the impact of mind–body approaches on sleep and psychological outcomes [44]. At the same time, the workshops highlighted inherent relational asymmetries that can arise within caregiving dyads. In some instances, caregivers were more vocal or confident during structured discussions and decision-making activities, reflecting well-documented power dynamics in dementia care contexts. To address this, we employed inclusive strategies such as directing questions explicitly to people living with dementia, using smaller dyadic breakout discussions, and inviting non-verbal or experiential forms of input (e.g., demonstrating or selecting preferred practices rather than relying solely on verbal explanation). These approaches helped ensure that the perspectives of people with dementia were actively elicited and respected, consistent with PPI principles and ethical guidance.

Recognising and actively managing such relational dynamics was integral to the co-design process and informed the final structure of DREAM. This reflexive attention to power, voice, and participation underscores the importance of skilled facilitation in dyadic and participatory intervention development and reinforces the relational foundations upon which DREAM is built.

### 4.2. Integrating Cultural Adaptation and Dyadic Mind–Body Practices

DREAM’s cultural adaptations—linguistically and visually tailored materials, culturally resonant metaphors, and delivery in trusted community venues—reflect evidence that adapted psychosocial interventions enhance accessibility and engagement among diverse groups [45]. Consistent with structured guidance on cultural adaptation in dementia care [46], these adaptations were achieved through iterative stakeholder consultation, co-design, and pilot delivery across four ethnic communities.

In addition to its cultural grounding, DREAM differs in important ways from previously published Tai Chi–based and mind–body interventions for people with dementia. Existing programmes, including the TACIT (TAi ChI for people with demenTia) intervention, have primarily focused on balance, postural control, and general wellbeing, often delivered either individually or with limited caregiver involvement. While TACIT demonstrated the feasibility and acceptability of Tai Chi for people with dementia—and highlighted challenges such as reliance on static instructional materials that may hinder adherence [47,48]—DREAM extends this work by embedding Tai Chi within a dyadic, caregiver-supported framework and by targeting sleep-related mechanisms rather than physical function alone.

Furthermore, unlike most Tai Chi programmes in dementia care, DREAM integrates brief, structured mindfulness practices alongside simplified Tai Chi–based movements within a single, coherent intervention. This integration is theoretically informed and purpose-driven: mindfulness practices were selected to reduce pre-sleep cognitive and emotional arousal, while Tai Chi movements were adapted to promote physiological relaxation and autonomic settling, thereby supporting sleep onset and continuity. Joint mindfulness interventions have shown promise in enhancing emotional regulation and dyadic attunement in dementia–caregiver pairs [49], but such approaches have rarely been combined systematically with movement-based practices or explicitly aligned with sleep outcomes.

More recent multimodal, therapist-led sleep interventions incorporating mind–body elements have demonstrated feasibility and acceptability [50]. However, these interventions typically emphasise clinical delivery and symptom management rather than culturally embedded, community-based implementation. In contrast, DREAM is explicitly designed as a culturally responsive, community-delivered programme, with its content and structure mapped to behavioural determinants using COM-B and behaviour change techniques. Together, this positions DREAM as a conceptually and clinically distinct intervention—one that builds on prior Tai Chi and mindfulness research while offering a novel, dyadic, and sleep-focused contribution to dementia care.

### 4.3. Behavioural Objectives and Change Mechanisms

A central contribution of DREAM lies in its explicit and systematic specification of behavioural objectives and mechanisms of action using the COM-B model and the TDF. Together, these frameworks enabled precise identification of the key determinants shaping engagement—capability, opportunity, and motivation—and informed the development of clearly defined performance objectives, including establishing dyadic bedtime routines, integrating mindfulness and Tai Chi into daily life, reducing night-time stress, and sustaining caregiver-supported home practice.

Importantly, DREAM differs from existing Tai Chi–based interventions in dementia care through its purpose-driven behavioural architecture. Tai Chi movements were not selected to optimise balance, strength, or general fitness, as is common in many programmes, but were deliberately chosen and adapted to target sleep-related mechanisms, including autonomic calming, attentional settling, and physiological downregulation. Movements were simplified, predominantly seated, and introduced through a progressive, dementia-sensitive sequence with repetition and scaffolding to minimise cognitive load and support learning over time.

DREAM also integrates brief, portable mindfulness practices—such as the body scan and 3 min breathing space—within a unified mind–body framework rather than delivering mindfulness and movement as parallel or standalone components. These practices were specifically selected to reduce pre-sleep cognitive and emotional arousal, while the Tai Chi-based movements support somatic relaxation and bodily readiness for sleep. This integrated approach creates a coherent pathway linking cognitive, emotional, and physiological regulation in relation to sleep.

A further distinguishing feature is the programme’s emphasis on caregiver-mediated practice. The dyadic structure positions the caregiver not only as a participant but as an active facilitator of routines and home practice, strengthening relational safety, reducing caregiver burden, and supporting habit formation—factors known to contribute to more stable and less disrupted sleep within dementia–caregiver dyads.

All behavioural objectives were systematically mapped to intervention functions (e.g., education, training, modelling, enablement, environmental restructuring) and to specific behaviour change techniques (BCTs), including action planning, habit formation, self-monitoring, prompts and cues, graded practice, and social support. This explicit mapping enhances transparency around how and why the intervention is expected to work, supporting theoretical precision, replicability, and implementation readiness [51,52,53,54]. By aligning behavioural design with emerging guidance from implementation science [55,56], DREAM offers a conceptually robust and practically actionable model for advancing non-pharmacological, sleep-focused interventions in dementia care.

### 4.4. Practical Outputs and Translational Potential

The DREAM development process resulted in a structured, ready-to-deliver non-pharmacological intervention that can be readily understood and utilised by clinicians managing sleep disturbance in people with dementia. The programme is supported by a comprehensive package of resources, including a Facilitator Manual, participant practice guides, home-practice logs, and feedback and monitoring tools, all culturally adapted to enhance accessibility across diverse communities.

From a clinical perspective, DREAM offers several features that align with common practitioner priorities. The eight-week, manualised structure allows for graded skill development while remaining time-limited and feasible within community care contexts. All Tai Chi-based movements are simplified, predominantly seated, and adaptable, supporting safety and minimising physical and cognitive burden for people with dementia. The dyadic format actively engages caregivers, a key consideration for clinicians seeking interventions that reduce caregiver strain while supporting adherence and continuity of care.

A clearly articulated logic model links behavioural determinants, intervention functions, and intended outcomes, providing transparency around mechanisms of action and supporting fidelity, replication, and evaluation. Positioned within community settings and voluntary sector delivery, DREAM aligns with contemporary models of dementia care, including social prescribing and community referral pathways, offering clinicians a low-risk, scalable alternative or adjunct to pharmacological management of sleep disturbance.

### 4.5. Strengths and Limitations

Strengths include the systematic use of IM, integration of behavioural theory and cultural adaptation, and multi-stage co-design involving diverse stakeholders. However, DREAM has not yet undergone formal feasibility or efficacy testing, requiring future evaluation of acceptability, fidelity, and effectiveness. While development involved participants from White British, Caribbean, Chinese, and South Asian communities, the sample in a single city in UK limits generalisability, and further work is needed to ensure accessibility for broader and intersectional groups. Voluntary participation may also introduce selection bias toward individuals with greater interest in mind–body practices. Scaling up will require structured facilitator training and fidelity procedures. Despite these limitations, DREAM provides a robust foundation for feasibility and implementation research and represents a culturally attuned, theoretically grounded approach to sleep and wellbeing in dementia care.

### 4.6. Implications for Research and Practice

The next phase of work will advance DREAM to Intervention Mapping Stages 5 and 6, focusing on implementation planning and evaluation within real-world care systems. During Stage 5, a detailed implementation strategy will be developed to address key considerations relevant to clinical and service contexts, including intervention fidelity, sustainability, workforce capacity, and integration across community and healthcare settings. A central component of this phase will be the development of a structured, theory-informed facilitator training programme, recognising that the effectiveness of culturally responsive and dyadic interventions depends heavily on delivery style and relational competence.

Planned facilitator training will include orientation to the DREAM logic model and behavioural change principles underpinning the intervention, alongside practical training in dementia-sensitive communication, cultural humility, and strategies for managing dyadic dynamics within group settings. Training will also address how facilitators can adapt pacing, language, and guidance to accommodate cognitive variability while preserving fidelity to core intervention components. To support scalability and sustainability, this training will be delivered through a train-the-trainer model, equipping community practitioners and voluntary sector staff to deliver DREAM with appropriate supervision, reflective practice, and fidelity monitoring. This approach is intended to reduce reliance on specialist facilitators and facilitate integration within established referral pathways, including memory services, primary care, and social prescribing.

Stage 6 will employ a Hybrid Type 3 feasibility design [57], prioritising the evaluation of implementation processes—such as acceptability, cultural fit, facilitator adherence, and delivery fidelity—while also examining preliminary clinical outcomes relevant to end-user clinicians, including sleep quality, caregiver wellbeing, and dyadic engagement. By addressing both implementation and early outcome indicators, this phase will generate evidence directly applicable to service planning and clinical decision-making.

In the longer term, the DREAM framework may have relevance beyond dementia care. Its emphasis on dyadic engagement, low-risk mind–body practices, and sleep-focused mechanisms suggests potential applicability to other neurodegenerative or chronic conditions commonly encountered in clinical practice—such as Parkinson’s disease or stroke—where sleep disturbance and caregiver wellbeing are similarly interconnected.

## 5. Conclusions

The DREAM programme demonstrates the feasibility and value of using Intervention Mapping to co-develop a dyadic, culturally adapted mind–body intervention for older adults with dementia and their caregivers. Grounded in behavioural theory, cultural adaptation, and community partnership, DREAM represents a robust model for non-pharmacological sleep intervention that integrates the biological, psychological, social, and cultural dimensions of care. Future feasibility and pilot evaluations will determine its acceptability, practicality, and potential for scalable implementation across diverse community settings. If successful, DREAM has the potential to offer a safe, accessible, and empowering non-pharmacological pathway to improving sleep for people living with dementia—and, crucially, for those who care for them.

## Figures and Tables

**Table 1:** Steps and tasks undertaken in DREAM intervention development (Intervention Mapping Stages 1–4).

Intervention Mapping Step	Key Tasks in the Development of DREAM
Stage 1: Needs assessment	(1) Stakeholder Engagement: Convened a diverse working group including dementia–caregiver dyads, PPI contributors, mind–body practitioners, community leaders, and clinicians. (2) Needs Assessment: Combined focus groups, stakeholder consultations, and a scoping review to identify sleep-related needs, preferences, and contextual enablers/barriers. (3) Outcome Prioritisation: Used workshops to identify sleep, dyadic engagement, and cultural acceptability as primary outcomes. (4) Behavioural Diagnosis: Applied BCW/COM-B to map determinants of engagement across capability, opportunity, and motivation. (5) Problem Analysis: Synthesised data into a needs statement informing Stage 2 outcome and change objective development.
Stage 2: Programme outcomes and change objectives	(1) Define Programme Outcomes: Agreed on core outcomes—improved sleep quality, enhanced caregiver wellbeing, and strengthened dyadic engagement. (2) Specify Performance Objectives: Identified target behaviours for people with dementia, caregivers, and facilitators to achieve desired outcomes. (3) Identify Determinants: Mapped modifiable barriers and facilitators using COM-B and TDFs across capability, opportunity, and motivation domains. (4) Develop Change-Objective Matrix: Linked determinants to intervention functions and behaviour change techniques to guide DREAM intervention design.
Stage 3: Methods and practical applications	(1) Collaborative Co-Design: Co-developed candidate intervention content and delivery formats through workshops with PPI contributors, caregivers, practitioners, and community representatives. (2) Technique Selection and Application: Identified and mapped theory-informed BCTs (e.g., action planning, habit formation, modelling, environmental restructuring) into culturally and contextually appropriate applications. (3) Logic Model Development: Specified and integrated the intervention’s logic model, linking behavioural determinants, selected techniques, and anticipated outcomes.
Stage 4: Programme design and materials	(1) Programme Development: Finalised programme scope, weekly themes, and session structure; produced culturally adapted facilitator and participant materials, including manuals, activity guides, and practice resources. (2) Iterative Refinement: Reviewed and refined materials with the working group, PPI contributors, and community stakeholders; pre-tested selected activities for clarity and cultural fit. (3) Implementation Supports: Developed delivery tools (e.g., session checklists, feedback forms, outcome measures) to ensure implementation fidelity, engagement tracking, and evaluative readiness.

**Table 2:** Matrix: Performance Objectives, Determinants, Change Objectives, and Mappings.

Performance Objective	Determinant (COM-B/Selected TDF Constructs)	Change Objective	Intervention Functions (BCW)	Example BCTs
Establish consistent dyadic bedtime routines	Capability (Knowledge, Skills); Opportunity (Social influences—caregiver support; Environmental context and resources)	Increase caregiver knowledge and skills to guide and support bedtime routines and create an enabling home environment.	Education; Training; Environmental restructuring; Enablement	Instruction on how to perform the behaviour; Action planning; Prompts/cues; Social support (practical); Restructuring the physical environment
Integrate mindfulness or gentle Tai Chi into daily practice	Capability (Skills, Memory/attention/decision processes); Motivation (Beliefs about capabilities; Reinforcement)	Enhance dyad skills and confidence to practise safely and regularly, with graded steps and reminders.	Training; Modelling; Enablement	Demonstration of the behaviour; Graded tasks; Habit formation; Prompts/cues; Feedback on behaviour
Reduce night-time stress through relaxation techniques	Motivation (Beliefs about consequences, Emotion); Opportunity (Social support)	Promote positive outcome expectancies and strengthen caregiver support for relaxation practices before bedtime.	Education; Persuasion; Enablement; Modelling	Credible source; Re-attribution; Social support (emotional); Demonstration of the behaviour; Behavioural practice/rehearsal
Maintain adherence to dyadic home practice	Motivation (Goals, Intentions); Opportunity (Environmental context and resources; Social influences)	Foster motivation and provide culturally tailored resources and schedules to support sustained home practice.	Enablement; Incentivisation; Environmental restructuring	Goal setting (behaviour); Review behaviour goals; Self-monitoring of behaviour; Prompts/cues; Rewards contingent on behaviour

**Table 3:** Intervention Priorities and COM-B Factors Among 12 Dementia–Caregiver Dyads (N = 24).

	White British (n = 6)	Caribbean (n = 6)	Chinese (n = 6)	South Asian (n = 6)	Overall (N = 24)
**Priority Areas**					
Sleep quality	6 (100%)	6 (100%)	5 (83%)	5 (83%)	22 (92%)
Dyadic engagement	4 (67%)	6 (100%)	5 (83%)	5 (83%)	20 (83%)
Cultural acceptability	2 (33%)	4 (67%)	6 (100%)	5 (83%)	17 (71%)
Stress management	4 (67%)	2 (33%)	4 (67%)	4 (67%)	14 (58%)
Caregiver wellbeing	4 (67%)	2 (33%)	4 (67%)	3 (50%)	13 (54%)
Sustainability of home practice	2 (33%)	4 (67%)	4 (67%)	4 (67%)	14 (58%)
**COM-B Domains—Capability**					
Cognitive load	4 (67%)	4 (67%)	4 (67%)	5 (83%)	17 (71%)
Physical limitations	4 (67%)	4 (67%)	3 (50%)	4 (67%)	15 (63%)
Unfamiliar with mind–body practices	2 (33%)	2 (33%)	5 (83%)	5 (83%)	14 (58%)
**COM-B Domains—Opportunity**					
Transport difficulties	2 (33%)	2 (33%)	4 (67%)	5 (83%)	13 (54%)
Limited trusted venues	2 (33%)	2 (33%)	4 (67%)	4 (67%)	12 (50%)
Competing responsibilities	4 (67%)	2 (33%)	2 (33%)	3 (50%)	11 (46%)
Cost concerns	2 (33%)	2 (33%)	4 (67%)	2 (33%)	10 (42%)
Language barriers	0 (0%)	1 (17%)	4 (67%)	3 (50%)	8 (33%)
Caregiver involvement essential	4 (67%)	6 (100%)	6 (100%)	5 (83%)	21 (88%)
**COM-B Domains—Motivation**					
Cultural meaning (facilitator)	4 (67%)	6 (100%)	6 (100%)	6 (100%)	22 (92%)
Perceived dyadic benefit	4 (67%)	4 (67%)	5 (83%)	5 (83%)	18 (75%)
Stigma (barrier)	0 (0%)	2 (33%)	1 (17%)	2 (33%)	5 (21%)
Scepticism	0 (0%)	2 (33%)	2 (33%)	2 (33%)	6 (25%)

**Table 4:** Weekly Programme Outline.

Session	Topics	Group Content	Mindfulness Component	Tai Chi Component
1	Awakening the Senses and Gentle Opening	Participants were introduced to mindfulness through the five senses exercise (paying attention to sights, sounds, smells, tastes, and touch) and a body scan that guided awareness through different parts of the body to release tension. The first Tai Chi sequence, Opening and Repulse Monkey, focused on smooth, coordinated arm movements and gentle shifting of weight to promote grounding and relaxation.	Five senses exercise; Body scan	Opening + Repulse Monkey
2	Awareness of Seeing and Breathing	After sharing reflections from home practice, participants explored mindful seeing (noticing visual details with curiosity) and mindful breathing (observing the breath without changing it). The Tai Chi sequence Brush Push built on earlier movements, encouraging flow and rhythm to synchronise breath with motion.	Sharing of homework; Mindful seeing; Mindful breathing	Continued: Brush Push
3	Listening with Awareness	This session deepened sensory mindfulness through mindful listening, helping participants tune into sounds around them and within the dyad relationship. A brief body scan reinforced body awareness. The Tai Chi sequence Part the Wild Horse’s Mane emphasised graceful arm extensions and balance, symbolising openness and release.	Sharing of homework; Mindful listening; Body scan	Continued: Part the Wild Horse’s Mane
4	Mindfulness of Inner Weather	Participants used the “weather forecast” metaphor to describe emotions (“sunny,” “windy,” or “stormy”) and recognise feelings without judgment. The mindfulness practice returned to mindful breathing to steady attention. The Tai Chi sequence Wave Hands Like Clouds introduced slow and circular hand motions, supporting relaxation and flow in a sitting position.	The weather forecast; Sharing of homework; Mindful breathing	Continued: Wave Hands Like Clouds
5	Using the Breath to Reset	Participants revisited breathing as a resource for calming and centring attention, practising the 3 min breathing space technique—a short mindfulness pause that can be used during moments of stress. The Tai Chi sequence Rooster Stands on One Leg was adapted as a seated balance exercise, in which participants gently raised one knee at a time to promote stability, coordination, and concentration while ensuring safety and accessibility.	Sharing of homework; Using your breath; 3 min breathing space	Continued: Adapted Rooster Stands on One Leg
6	Directing and Sustaining Attention	Mindfulness practice focused on focusing attention and moving it deliberately, training mental flexibility and awareness of shifting thoughts. The 3 min breathing space was practised again as a portable coping tool. The Tai Chi movement Kick with Heel encouraged gentle leg strength and coordination, adapted for seated practice.	Sharing of homework; Focusing attention and moving it; 3 min breathing space	Continued: Kick with Heel
7	Mindful Communication and Connection	The focus turned to mindful listening to each other, promoting empathetic communication and shared awareness within the dyad. The 3 min breathing space was used collaboratively, encouraging caregivers and people with dementia to practise together. The Tai Chi form Grasp the Peacock’s Tail combined flowing sequences from previous weeks, symbolising harmony and balance between partners.	Sharing of homework; Mindful listening to each other; 3-min breathing space	Continued: Grasp the Peacock’s Tail
8	Consolidation and Closure	The final session revisited the body scan for grounding and reflection, followed by a group sharing of experiences and intentions for continuing home practice. The Tai Chi sequence Cross Hands and Closing Form provided a calm, circular movement to conclude the course, integrating the themes of balance, connection, and stillness.	Body scan; Conclusion sharing	Continued: Cross Hands

## Data Availability

The data presented in this study, including intervention materials where appropriate, are available on request from the corresponding author. The data are not publicly available due to organizational and ethical restrictions.

## Abbreviations

The following abbreviations are used in this manuscript:

BCT	Behaviour change techniques
BCW	Behaviour Change Wheel
COM-B	Capability, Opportunity, Motivation → Behaviour
DREAM	Dyadic Resilience, Engagement, Awareness & Mind–body intervention
DREAM START	Dementia Related Manual for Sleep; Strategies for Relatives
IM	Intervention Mapping
MRC	Medical Research Council
PPI	Patient and Public Involvement
TACIT	TAi ChI for people with demenTia
TDF	Theoretical Domains Framework
VCSE	Voluntary, Community and Social Enterprise
